# Drug repurposing in traditional Chinese medicine: from empirical wisdom to modern therapeutic strategies

**DOI:** 10.3389/fphar.2025.1631727

**Published:** 2025-07-31

**Authors:** Yuan Wu, Jiarong Huang, Jiajun Guo, Wanming Lian, Maorong Suo

**Affiliations:** College of Jiyang, Zhejiang A&F University, Zhuji, China

**Keywords:** Traditional Chinese medicine, drug repurposing, systems biology, network pharmacology, precision medicine, translational research

## Abstract

Traditional Chinese medicine (TCM), with its multi-component and multi-target nature, offers rich potential for drug repurposing. Advances in systems biology, computational modeling, and high-throughput technologies have enabled systematic analysis of TCM mechanisms, facilitating in the identification of active ingredients, target interactions, and synergistic effects. However, most existing reviews focus on individual methods rather than providing an integrative translational strategy tailored to the complexity of TCM. This review addresses that gap by proposing a stage-based framework that combines mechanism analysis, preclinical validation, and clinical translation. We highlight key methodologies such as network pharmacology, multi-omics, molecular docking, and phenotypic screening, and discuss how they can be synergistically applied. Emerging technologies including AI, big data, 3D bioprinting, and organoid models are evaluated not only for their utility but also through critical analysis of their limitations in capturing TCM’s holistic principles. By bridging traditional knowledge with modern biomedical innovation, this review offers a novel roadmap for accelerating evidence-based TCM repurposing. The proposed integrative strategy supports more reproducible, mechanistically grounded, and globally relevant applications of TCM in modern drug development.

## 1 Introduction: the potential of TCM in drug repurposing

Traditional Chinese medicine (TCM), characterized by its multicomponent herbal formulations and polypharmacological mechanisms, is grounded in millennia of empirical clinical practice. This characteristic offers potential for expanding the clinical indications of TCM formulas. Fufang Biejia Ruangan Pill (FBRP) is the first TCM formula approved in clinic and is mainly applied for an-ti fibrosis treatment. Zhang et al. found this formula has the potential for treating liver cancer through PI3K/AKT/NF-κB signaling pathway (Zhang et al., 2020). Buzhong Yiqi Decoction (BZYQD) is another classical TCM formula for strengthen the immune system, and recent study has demonstrate its effectiveness in treating polycystic ovary syndrome (PCOS). The overall effectiveness rate was 67.7% (Hu et al., 2024). It was also suggested that BZYQD combined with other drugs could significantly improve lung function in patients with chronic obstructive pulmonary disease (COPD) (Yi-Ling et al., 2020).

TCM formulas have various active compounds, and the therapeutic effect of TCM depends on complex interaction of these compounds in human bodies. When TCM formulas were expected to expand indications, several challenges appeared due to the complexity of TCM. Firstly, we need to elucidate the targets and molecular mechanisms of TCM, but it is hard to solve this issue via single-omics approaches (Xu et al., 2021), so a combined multi-omics strategy is necessary to be used. For example, Lin et al. has already combine multi-omic analysis including network pharmacology to elucidate the multi-pathway mechanisms of Huiyang Shengji Decoction (HYSJD) (Lin et al., 2025). Secondly, the preclinical evaluation system for TCM repurposing is still deficient. Several issues including non-standardized production processes, weak quality control systems, and inadequate toxicological evaluation are not been solved (Berdigaliyev and Aljofan, 2020; Hua et al., 2025). Thirdly, translational process of TCM repurposing also face various difficulties, such as inconsistent regulatory standards, and incompatibility between traditional Western trial designs and the holistic intervention characteristics of TCM (Hua et al., 2025).

**FIGURE 1:**
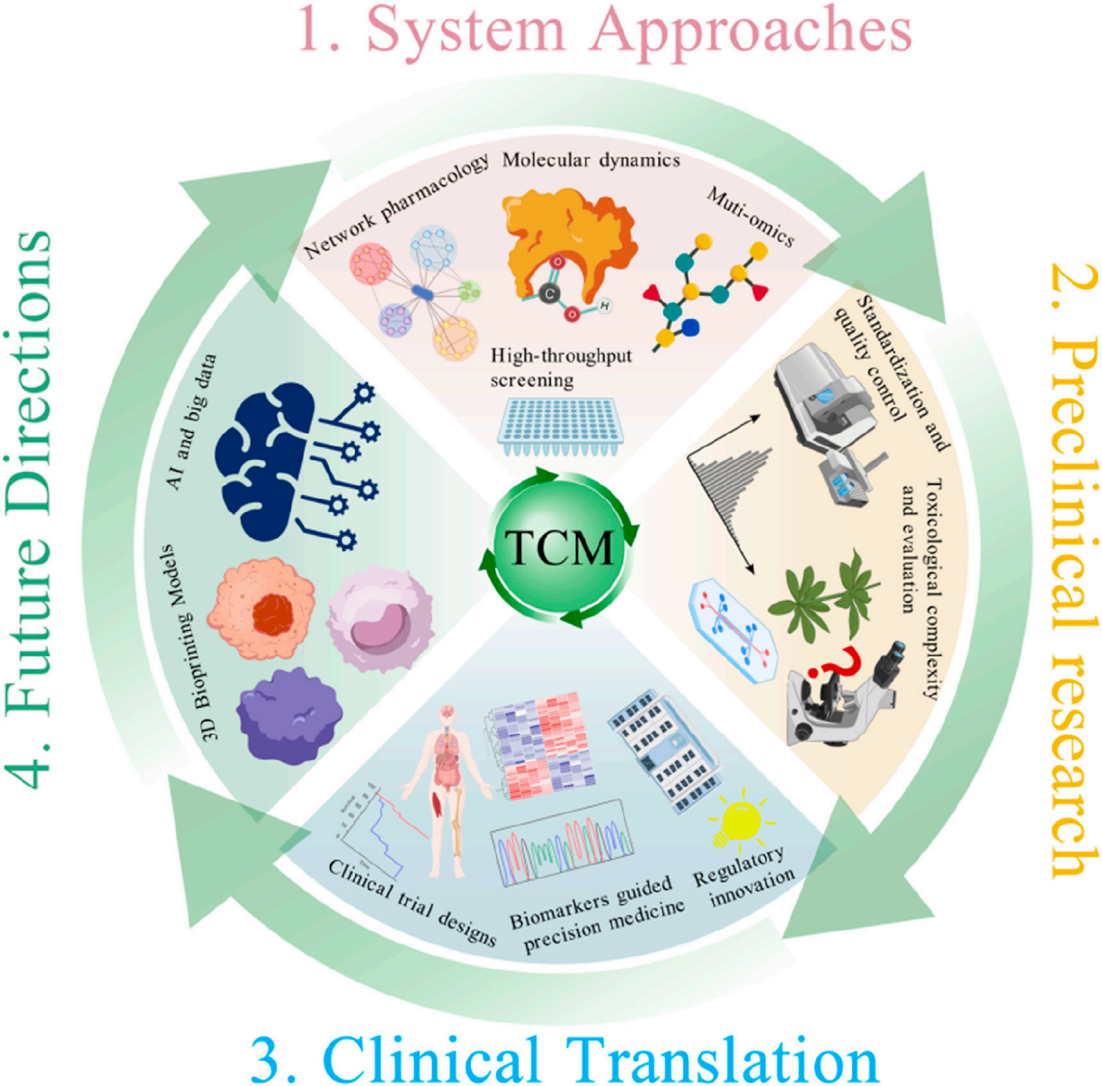
Schematic framework of TCM repurposing integrating system approaches, preclinical research, and clinical translation.

In recent years, the integration and application of various technologies, such as network pharmacology (Zhang et al., 2019b), multi-omics technology (Wang et al., 2021) and computational models (Jiao et al., 2021), have provided new directions for elucidating the mechanisms of TCM formulas. The combined application of these techniques enables the systematic construction of “ingredient-target-pathway-phenotype” interaction networks, revealing the multidimensional mechanisms underlying TCM actions. However, previous studies and reviews primarily focus on elucidating the basic mechanisms of TCM through these techniques and lack a systematic framework that advances mechanistic research toward clinical applications of TCM. This review aims to fill that gap by offering a unified, translational strategy that aligns modern systems technologies with TCM’s polypharmacology. Herein, we systematically summarize current progress in TCM-based drug repurposing research, with a focus on multi-level systems biology methodologies, challenges and innovative strategies in preclinical development, and clinical translational designs adapted to TCM characteristics. Furthermore, we explore how emerging technologies such as artificial intelligence, big data analytics, and 3D bioprinting could accelerate the modernization and internationalization of TCM repurposing. Importantly, we propose an innovative translational model for TCM drug repurposing that systematically integrates multidisciplinary approaches across the whole drug development pipeline. There are three key stages in this model including mechanism analysis based on system approaches, preclinical research and clinical translation (Figure 1) (Jiang et al., 2025). By directly addressing the complexities and challenges of TCM, this model aims to provide a comprehensive and practical roadmap to accelerate the development and clinical application of TCM repurposing. Notably, despite TCM repurposing contributes to TCM modernization, they are different concepts. The fundamental objective of TCM repurposing is exploring new indications for the existing TCM formulas or their active ingredients driven by mechanistic insights, omics data, and translational frameworks. While TCM modernization is a broader concept aiming to improve the scientific rigor, normative and international recognition of TCM for its sustainable development (Hua et al., 2025). TCM modernization usually includes quality control, mechanistic elucidation, regulatory adaptation, as well as clinical evidence building. Zhang et al. emphasized that long-term interdisciplinary efforts are required for TCM modernization (Zhang et al., 2019a). In this review, we highlight TCM repurposing as an important strategic component in TCM modernization through the combination of systems biology methodologies, innovative preclinical strategies, and clinically translational frameworks.

## 2 Study design and methods

**FIGURE 2:**
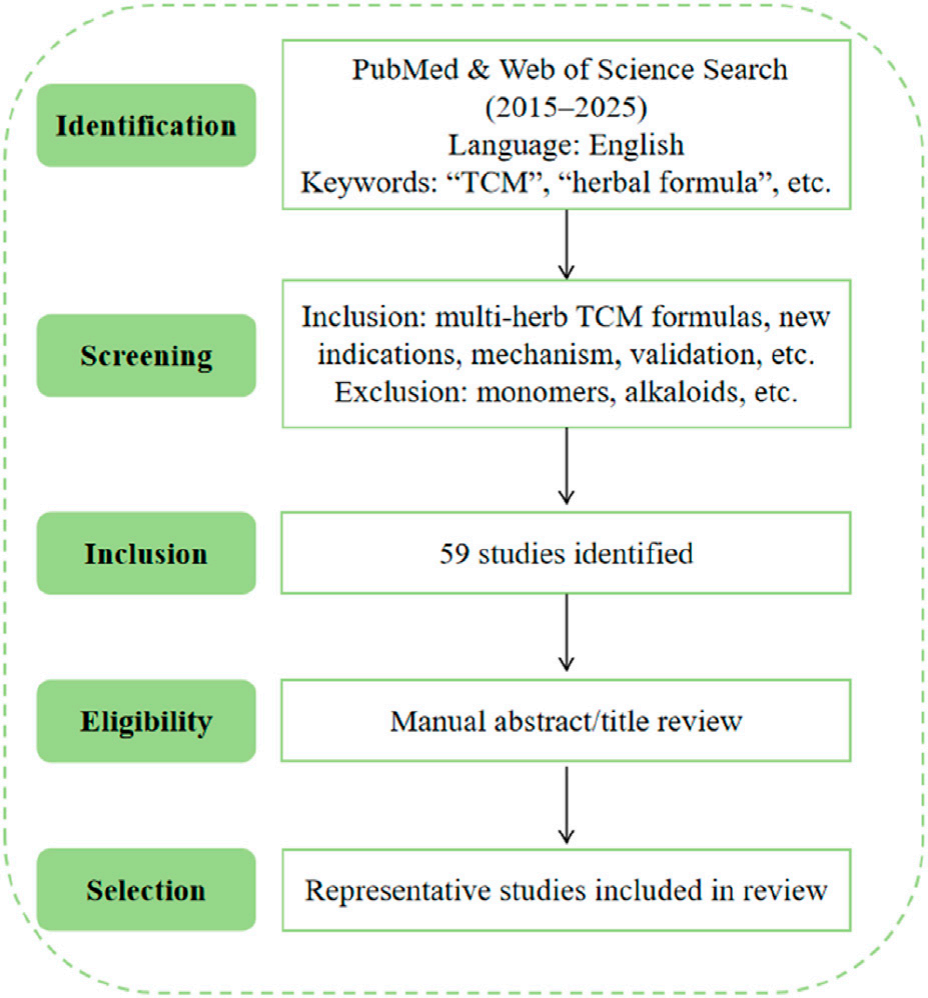
Literature selection workflow for TCM-based drug repurposing (2015–2025).

This review included original research studies on TCM–based drug repurposing, retrieved from English-language articles in the PubMed and Web of Science databases between 2015 and 2025. The search focused on multi-herb TCM formulas applied to new indications, with emphasis on molecular mechanisms, target validation, and preclinical or clinical translational evidence. Search terms included combinations of “traditional Chinese medicine”, “herbal formula”, “drug repurposing”, “new indications”, “mechanism”, “target”, “validation”, and related phrases. Studies focused solely on isolated monomers, alkaloids, or single compounds were excluded. In total, 59 original research articles were identified through this search strategy. Titles and abstracts were screened manually for relevance to drug repurposing mechanisms in TCM. Among these, representative studies were selected based on scientific rigor and relevance to the thematic framework of this review. The literature selection workflow of the study is displayed in Figure 2.

## 3 Systems approaches for TCM-based drug repurposing

The first step in TCM repurposing is to clarify the active components, pharmacology, and molecular mechanisms of traditional formulas. Over the past decades, with advancements in biotechnology, modern research has established a relatively complete systems biology framework for deciphering the multi-target pharmacological mechanisms of TCM. This framework primarily encompasses network pharmacology, molecular docking, multi-omics analysis, and high-throughput screening. This section will primarily explore the roles of these strategies in the study of multi-target pharmacological mechanisms of TCM, as well as their significant value in promoting the expansion of TCM indications. The representative studies of TCM repurposing are summarized in the Table 1.

**TABLE 1:** Representative studies of TCM repurposing.

Formula	Original indication	Repurposed indication	Key mechanisms	Evidence level	Ref.
Buzhong Yiqi Decoction (BZYQD)	Spleen Qi deficiency	PCOS; COPD	Kisspeptin-GPR54 pathway; IL-6/TNF-α	Clinical and *in vivo*	Hu et al. (2025), Yi-Ling et al. (2020)
Fufang Biejia Ruangan Pill (FBRP)	Anti-fibrosis	Hepatocellular carcinoma	PI3K/AKT/NF-κB signaling	*In vitro* and pathway analysis	Zhang et al. (2020)
Liuwei Dihuang Wan	Kidney yin deficiency	Diabetes, osteoporosis	PI3K/AKT/FOXO, VEGF	*In vivo*	Lu et al. (2022)
Xuefu Zhuyu Decoction	Coronary heart disease	Pulmonary hypertension	COPD-related genes and inflammation	Network pharmacology and *in vitro*	Yu et al. (2024)
Bazibu Shen Capsule	Male reproductive health	Alzheimer’s disease	ERK1/2/NF-κB, GSK-3β/β-catenin	*In vitro* and prediction	Wang et al. (2023)

### 3.1 Network pharmacology: unraveling multi-target mechanisms for TCM

Network pharmacology has emerged as a pivotal methodology for deconstructing the multi-target, multi-pathway mechanisms underlying TCM efficacy. By constructing “herb-compound-target-disease” interaction networks, this approach transforms traditional therapeutic principles into mechanistically testable hypotheses (Li and Zhang, 2013; Zhang et al., 2019b). Key analysis processes of this approach include data mining, network construction, as well as functional prioritization. Relevant tools are listed in Supplementary Table S1.

During the past decade, various studies have demonstrated that network pharmacology plays a significant role in expanding the clinical indications of TCM as well. For example, as a classical TCM formula, Bazibu Shen Capsule (BZBS) is originally used for treating male reproductive system diseases in clinic. However, through network pharmacology analysis combined with *in vitro* experimental verification, Wang et al. revealed that it exerts potential therapeutic effects on Alzheimer’s disease by multi-target regulation of the ERK1/2/NF-κB and GSK-3β/β-catenin signaling pathways (Wang et al., 2023). Another example is Jiawei Buzhong Yiqi Decoction, which is originally used for treating diseases related to spleen-stomach qi deficiency. Recently, Hu et al. reported that it exerts therapeutic effects on obesity-related polycystic ovary syndrome (PCOS) by regulating the kisspeptin-GPR54 pathway and the production of sex hormone-binding globulin (SHBG) (Hu et al., 2025). Liuwei Dihuang Wan, a classic formula for nourishing yin and tonifying the kidney, is primarily used to treat kidney yin deficiency. Lu et al. found that it can treat diabetes and osteoporosis by acting on the PI3K/AKT/FOXO and VEGF signaling pathways (Lu et al., 2022). Additionally, Xuefu Zhuyu Decoction, clinically used for treating coronary heart disease and other diseases, has been shown to treat pulmonary hypertension by targeting genes associated with chronic obstructive pulmonary disease (COPD), ischemia, and myocardial infarction. (Yu et al., 2024). Within our proposed systematic framework, network pharmacology acts as a critical tool for virtual screening and initial mechanism prediction, forming the early stage of TCM repurposing pipelines. Nevertheless, these databases have limitations and challenges. Most data from the database rely on predictive algorithms and literature mining with limited experimental validation. According to our analysis of 59 reviews, 63% of studies lack a complete experimental validation chain, with 41% relying solely on computational predictions and 22% limited to *in vitro* assays. These data were calculated based on the 59 studies obtained in Study design and method, and the titles of these studies were listed in Supplementary Table S2.

### 3.2 Molecular docking and molecular dynamic simulation: predicting poly-pharmacological interactions in TCM

Molecular docking and molecular dynamics (MD) simulation are pivotal computational techniques in drug discovery, crucial for predicting new targets of TCM bioactive compounds (Jiao et al., 2021; Saikia and Bordoloi, 2019). Docking estimates the optimal binding conformation of a ligand to a target protein by simulating intermolecular interactions and scoring binding affinities, while MD simulations model ligand-protein interactions under physiological conditions, capturing conformational dynamics, binding stability, and free energy landscapes. These techniques have successfully elucidated multi-target mechanisms of various TCM formulations. For example, combined network pharmacology and docking revealed how Xianlingubao might treat diabetic osteoporosis (Yan et al., 2024). Similarly, molecular docking has shown that flavonoids (e.g., quercetin, kaempferol) and saponins (e.g., ginsenosides) can bind to key inflammatory enzymes (COX-2, iNOS) and metabolic regulators (PPAR-γ, AMPK) (Bastin et al., 2023; Li et al., 2023; Lin et al., 2022; Yasir et al., 2024). Subsequent MD simulations confirm the stability of these interactions, revealing persistent hydrogen bonding and favorable conformational dynamics under physiological conditions. The relevant tools are listed in Supplementary Table S3.

Together, despite molecular docking and MD simulations provide a robust framework to uncover poly-pharmacological mechanisms of TCM compounds, accelerating their transition into modern therapeutics. However, these methods cannot fully reflect the complex physiological environment because they are computational prediction tools. Moreover, simulations only reflect potential binding trends and cannot replace *in vitro* or *in vivo* experimental verification (Duan et al., 2025).

### 3.3 Integrative multi-omics approaches: bridging genomics, proteomics, and metabolomics

Multi-omics approaches, including genomics, transcriptomics, proteomics, and metabolomics, serve as powerful tools in modern biology. They provide key support for comprehensively understanding the multi-target mechanisms of TCM and exploring its new indications (Wang et al., 2021). Genomic analysis can deeply show the genetic reasons behind diseases. This helps find new possible indications to apply TCM. For example, genome-wide association studies (GWAS) have helped us better understand how ischemic stroke happens, especially about oxidative stress and nerve inflammation. Based on this, active substances from TCM like sesamin and baicalin have been rechecked. They were first used for anti-oxidation/lowering lipids and clearing heat/removing toxins. Now we know they can protect nerves well (Chen et al., 2020). Transcriptomic technologies can sensitively capture gene expression changes after TCM intervention, directly helping to clarify the new mechanisms of known formulas and promote their repurposing. For instance, previous study performed transcriptomics analysis and demonstrated that Dachaihu Decoction has the potential to treat sepsis-induced intestinal dysfunction via PI3K/AKT pathway. And the common application in clinic for Dachaihu Decoction is Shaoyang-Yangming syndromes (Huang et al., 2025).

Different with genomics and transcriptomics which showed the change of DNA/RNA level, proteomics and metabolomics display the changes in protein expression and metabolic flux. Kuai et al. used proteomics to analyze samples after treatment by Xiaoyin Granules, and found it was a candidate TCM for psoriasis through regulation estrogen signaling pathways as well as cholesterol metabolism (Kuai et al., 2020). Moreover, Astragalus membranaceus (AM), a well-known TCM, has been identified as a potential drug for hyperuricemia through analyzing metabolites in blood, urine, and fecal from hyperuricemic rats by metabolomics. Zhang et al. found that AM significantly increased lipid signaling molecules, hormone synthesis, unsaturated fatty acid absorption, as well as tryptophan metabolism (Zhang et al., 2022b).

During the past decade, the muti-omics techniques have made great progress due to the advancements of bioinformatics and analytical techniques. For example, the generation of Whole-genome sequencing (WGS) enables unbiased scanning of the entire genome and helps explore unknown aspects of TCM mechanisms (Caspar et al., 2020). Single-cell and spatial transcriptomics allow precise mapping of gene expression profiles across different cell types or spatial locations, aiding in understanding TCM’s role in tissue microenvironments (Gulati et al., 2025). Liquid chromatography-mass spectrometry (LC-MS), which offers higher sensitivity than using LC or MS alone, is effective for detecting metabolic and proteomic changes under TCM treatment (An et al., 2024). Nevertheless, the single application of multi-omics technologies still has certain limitations. Since omics data essentially reveals correlations rather than causal relationships, rigorous *in vivo* experiments are required to validate predicted targets and pathways, thereby establishing causal relationships in the mechanisms.

### 3.4 High-throughput screening: a modern tool driving drug repurposing in TCM

High-throughput screening (HTS) is not only a key technology in the first step of expanding indications for TCM, but also an essential tool in the early stages of drug development for systematically discovering active compounds and their novel therapeutic applications (Dove, 2003). Modern HTS techniques, such as ligand-binding assays and downstream signaling pathway analyses, enable the efficient identification of bioactive molecules. TCM is an important source of active molecules, thus, it has significant value to systematically excavate active components from TCM through modern HTS technologies for accelerating new drug discovery and mechanistic interpretation. For example, a G-protein-coupled receptors (GPCRs) platform which aimed to discover novel candidate drugs from TCM was developed by HTS (Bi et al., 2025). And GPCRs have been confirmed too be potential targets of various compounds derived from TCM (Zhu et al., 2022). What’s more, Liu et al. build a library whose compounds were obtained from 202 medicinal plants and fungi by HTS, aiming to find active components and elucidate their mechanisms (Zhang et al., 2015). High-content screening (HCS) is the advanced version of HTS, and it can not only find active compounds from TCM but also obtain phenotypes and function data of related diseases (Wang et al., 2019). Song et al. has utilized HCS to develop a near-infrared fluorescent substrate screening platform whose response is fast. Based on this platform, the study not only identified 5-methoxypsoralen (5-MP) from Psoralea corylifolia, but also demonstrated the mechanism of 5-MP on bone mineral density (BMD) (Song et al., 2025).

Despite the above research indicates the potential utilization of HTS and HCS in TCM drug repurposing, they still face multiple challenges. For example, the screened active compounds by HTS/HCS often lack clear target sites and molecular mechanisms, that limits further pharmacological research and clinical translation. Additionally, most current studies remain at the *in vitro* level, lacking validation from *in vivo* experiments. Therefore, the effective application of HTS/HCS requires further integration with omics analysis, target validation, and systematic pharmacological research (Guo et al., 2022).

## 4 Challenges and innovations in preclinical development of TCM repurposing

Preclinical research is the second stage in expanding new indications for TCM, with its primary objective being to translate the mechanistic research findings from the first stage into viable candidate drugs through rigorous safety/efficacy evaluations, standardization, quality control, and toxicological assessments. This chapter will focus on the key aspects of standardization and quality control faced by TCM during the expansion of new indications, as well as the application of advanced analytical technologies in component detection and stability assessment of TCM. Meanwhile, to address the toxicological challenges posed by the multi-component complexity of TCM, this chapter will also explore integrative safety assessment strategies.

### 4.1 Standardization and quality control: cornerstones of safety and efficacy

In the preclinical research for expanding TCM indications, standardization and quality control of medicinal materials must be strictly implemented to ensure the safety, efficacy, and reproducibility of studies. First, the geographical origin of TCM materials requires explicit documentation and systematic research, as different producing areas directly affect the content of active components and pharmacological properties. For example, Scutellaria baicalensis from Shanxi contains 23%–68% higher levels of baicalin, baicalein, and baicalone than those from other regions. Thus, Shanxi-produced Scutellaria baicalensis is clinically more suitable for treating antiviral respiratory diseases, while those from other regions are better suited for immune-related chronic diseases (Lv et al., 2024) Comprehensive databases established in recent years (e.g., ETCM v.2.0) effectively support the recording and tracing of geographical origins, facilitating origin traceability management to ensure the rationality and consistency of homonymous medicinal materials used for different indications under varying regional conditions (An et al., 2024; Zhang et al., 2023). Additionally, traditional processing techniques (Paozhi) of TCM materials must be unified and standardized, as different processes affect their pharmacological properties. For instance, raw rehmannia root (Sheng Dihuang) and processed rehmannia root (Shu Dihuang) are two forms of Rehmannia glutinosa (raw material without steaming vs steamed product), exhibiting distinct pharmacological effects. Sheng Dihuang primarily exerts heat-clearing effects through intact glycosides, while Shu Dihuang achieves blood-nourishing and yin-tonifying effects via Maillard reaction products (Xia et al., 2020). Similarly, databases like KampoDB systematically record these chemical transformations, effectively promotingpharmaceutics standardization (Sawada et al., 2018).

During the preclinical research of TCM repurposing, in addition to strengthening the geographical traceability of raw materials and standardized management of processing techniques, it is equally significant to introduce advanced analytical technologies to detect and quantify active components. Such technologies not only enable high-precision analysis of TCM’s complex component systems, but also dynamically assess the specific impacts of different processing and extraction methods on component structures and their biological activities. Currently, high-performance liquid chromatography (HPLC) and liquid chromatography-mass spectrometry (LC-MS) have become core tools in standardized research on TCM components. For example, Bergonzi et al. used HPLC to precisely quantify antioxidant components in Gardenia jasminoides (Bergonzi et al., 2012). Furthermore, Liu et al. integrated LC-MS and nuclear magnetic resonance (NMR) to systematically identify the structural and quantitative characteristics of 42 compounds in Chaihu Longgu Muli Decoction, and clarified their stability ranges under various processing conditions (Liu et al., 2024a).

### 4.2 Toxicological complexity: integrated safety assessment strategies

Preclinical toxicological research represents a critical step in ensuring drug safety and clinical feasibility. Owing to the multi-component and multi-target characteristics of TCM compounds, their preclinical toxicological studies face unique challenges. On one hand, interactions among multiple active components of TCM may lead to unpredictable toxic manifestations in new indications ((Wu et al., 2018). This primarily stems from insufficient mechanistic understanding of toxic targets in current research. For example, aristolochic acid is a natural toxic component found in certain TCM materials. It exhibits significant nephrotoxicity and carcinogenicity, but scientists have not fully identified the non-covalent binding proteins involved (Zhang et al., 2022a). On the other hand, the preclinical toxicity assessment of TCM lacks unified and standardized detection methods, such as inconsistent specific toxicity endpoints for drug-induced liver injury (DILI) (Sun et al., 2022).

To address these challenges, it is essential for researchers to propose integrative safety assessment strategies. First, systems toxicology provides a holistic framework for this strategy by mapping the perturbation of metabolic and inflammatory pathways induced by TCM in new indications (Liu et al., 2024b). Second, artificial intelligence-driven predictive models can not only predict the distribution of toxic effects by mining potential associations between TCM components and targets but also quantitatively analyze interactions among quality markers (Q-markers) (Ding et al., 2024). This strategy closely integrates computational prediction, multi-omics analysis, and biomimetic verification, establishing a scientifically sound foundation with clear mechanisms and controllable safety for toxicological research in TCM repurposing.

## 5 Translational research strategies: from bench to bedside

Translational research is the third stage in expanding new indications for TCM, succeeding the systematic studies on active components, pharmacological mechanisms, and safety assessments of TCM formulas. The goal of this stage is to translate the candidate drugs selected from stage 2 into clinic. In this chapter, we discuss the complementary clinical trial designs based on traditional clinical approaches, the biomarker-guided precision medicine as well as regulatory innovations in China.

### 5.1 Adapting clinical trial designs for TCM drug repurposing

Randomized controlled trials (RCT) is the golden standard for clinical trials of drug candidates, while several challenges were confronted when it is applied in clinical studies of TCM. On the one hand, the core principles for RCT are single-variable control and standardized intervention protocols, that is conflicting with the multi-component and multi-target nature features of TCM (Bothwell et al., 2016; You et al., 2025). On the other hand, TCM emphasizes individualized treatment and syndrome differentiation, that would violate the RCT principles of rigid randomization and blinding designs. Thus, these dilemmas led to incomplete results of TCM clinical studies such as non-alcoholic fatty liver disease (NAFLD) (Liang et al., 2021). Another TCM study also confirmed the existence of these dilemmas. FYTF-919 is a common and effective TCM formula in clinic for intracerebral hemorrhage therapy. However, a RCT across 26 hospitals, which is randomized, double-blind and placebo-controlled, demonstrated there was no significant difference between FYTF-919 and placebo in terms of functional recovery, survival rate, or quality of life at 90 days (p = 0.63) (Guo et al., 2024a). The above studies highlight that RCT cannot accurately reflect efficacy of TCM interventions when proper target population and therapeutic target are not stratified.

To resolve the defects of RCT in TCM repurposing, recently, Bayesian trial designs and real-world evidence (RWE) are put forward. Different with RCT, it is allowed to integrate prior knowledge in Bayesian trial designs, and the trial protocol is also allowed to be adjusted during the clinical trial (Carriger et al., 2016). Actually, Bayesian trial designs have already been used for clinical trials of TCM. For example, Zhang et al. combined the Bayesian designs into their TCM clinical trials aiming to evaluate the effectiveness of seven TCM injections for neonatal hypoxic-ischemic encephalopathy therapy. And the results have demonstrated the trial design is suitable and efficient (Zhang et al., 2024). Different with RCT, RWE is able to reflect drug performance in daily life, that suggests RWE can evaluate drug efficacy and safety on broader population. The feasibility and efficacy of RWE have already been demonstrated in clinical trials focusing various diseases including diabetic macular edema and atopic dermatitis (Abuabara et al., 2023; Gabrielle et al., 2023). The application of Bayesian trial designs and RWE provides more possibilities for TCM repurposing in clinic.

### 5.2 Precision medicine empowering TCM repurposing

Through bring in quantifiable and objective biomarkers, precision medicine provides a systematic solution for clinical research on TCM repurposing, and it also helps align TCM research with modern pharmaceutical development standards. Imaging-derived biomarkers have proven valuable in evaluating TCM efficacy and expanding new indications. Functional magnetic resonance imaging (fMRI) has shown that Bushen Capsule enhances brain activity by 12% in patients with cognitive impairment (Zhang et al., 2019b),magnetic resonance spectroscopy (MRS) found that liver fat content in nonalcoholic fatty liver disease (NAFLD) patients decreased by 25% after TCM treatment (Yuan et al., 2023); and radiomics analysis showed that specific TCM interventions can reduce tumor volume by 30% (Mall et al., 2016). These imaging biomarkers not only provide visual evidence of TCM efficacy but also offer a scientific basis for cross-disease indication expansion. At the molecular level, biomarkers are also deepening our understanding of TCM mechanisms. Cai et al. identified 16 gut microbiota-related biomarkers revealing Danshen’s mechanism in treating kidney failure (Cai et al., 2019). Another study showed that MKG reduced serum IgE levels by 40% in asthma patients through IL-4/IL-13 pathway-related biomarkers (Yu et al., 2017).

For safety and toxicity evaluation in TCM clinical application, precision medicine is also bringing in biomarkers for risk monitoring. For instance, Hu et al. screened hypoxanthine, Lysopc (P-16:0/0:0), and taurodeoxycholic acid via LC-MS as potential biomarkers for Polygonum multiflorum-induced liver injury (PMR-ILI) (Hu et al., 2023). What’s more, Guo et al. identified hemoglobin beta subunit (HBB) as a key biomarker for aconitine-induced cardiotoxicity, reflecting individual susceptibility (Guo et al., 2024b). However, TCM biomarker research remains in early stages, limiting its broad application and clinical translation for new indication development.

### 5.3 Regulatory innovation in China: the “Three-Combinations” policy enabling TCM repurposing

Besides novel technique applications in TCM repurposing, policy innovation in China is also play an essential role in facilitating the clinical translation of TCM. For example, the National Medical Products Administration (NMPA) proposed the “Three-Combinations” policy in 2021 which emphasizes the combination of TCM theory, “human experience” and clinical trials Under this regulatory, expansion of new indications for TCM formulas can be supported not only by RCTs, but also by RWE, observational studies as well as classical medical records. Moreover, adaptive trial designs and post-marketing evidence collection are encouraged in this policy, and the regulatory clearly supports TCM formula studies with repurposing potential (http://www.nmpa.gov.cn/). Thus, this policy allows a more adaptable pathway for new indication expansion of TCM. For example, under the guidance of the “Three-Combinations” policy, three formulas including Lianhua Qingwen, Jinhua Qinggan, and Xuebijing were successfully repurposed and approved in 2021 for COVID-19 therapy (approval number: XZXK-2021–121, NMPA). These are the first batch of TCM products evaluated under the new Category 3.2 registration pathway defined by the NMPA.

## 6 Future directions and multidisciplinary integration

### 6.1 AI and big data in TCM drug repurposing

AI and big data technologies are modernizing TCM repurposing. Machine learning enables toxicity prediction (Song et al., 2024), compound-target affinity analysis (Saikia and Bordoloi, 2019), and latent indication discovery from clinical records and classical texts (Zhou and Wang, 2014). Databases like SymMap, TCMID, and TCMSP support data-driven repurposing efforts (Song et al., 2024).

However, challenges remain: TCM’s polyherbal nature, multi-target actions, and systemic effects do not align with single-target modern paradigms (Liu et al., 2020). Dataset limitations, inconsistent quality, and lack of standardization constrain AI model performance (Hopkins, 2008). Moreover, current AI models have the risk of algorithmic bias. Most models are trained based on limited datasets, which are unable to cover the unique information structure of TCM, that may lead to overfitting and poor generalization ability. At the same time, clinical information of TCM is mostly in text form and lacks systematic organization, severely limiting the training effect of supervised learning models. To address this, priorities include expanding TCM databases (e.g., ethnopharmacology, multi-omics), developing high-fidelity *in vitro* systems, and integrating genomics, proteomics, and imaging to bridge TCM with precision medicine.

### 6.2 3D bioprinting and organoid models: systems-vetting platforms for preclinical TCM

As the preclinical validation core of our stage model, vascularized multi-organ chips (vMOCs) represent a paradigm shift from reductionist models. These technologies replicate human physiology more accurately than 2D cultures or animal models, specifically engineered for TCM’s multi-target complexity.

3D bioprinting and organoid technologies replicate human physiology more accurately than 2D cultures or animal models, offering ethical, cost-effective platforms for evaluating TCM. For instance, intestinal organoids revealed that Schisandrin C enhances barrier function via tight junction proteins in IBD (Kim et al., 2022). 3D-bioprinted inflammation models showed Danggui Niantong Decoction suppressed cytokines more effectively than 2D systems (Liang et al., 2024). More significantly, liver-gut co-cultures simulate enterohepatic metabolism, that is essential for modeling TCM formula pharmacokinetics where animal models fail.

Innovative solutions overcome TCM-specific barriers. For example, to address vascularization deficits in organoids, microfluidic perfusion systems now maintain nutrient gradients (Yang et al., 2025). Single-organ models cannot capture systemic effects, thereby gut-liver-brain MOC platforms is built to decode cross-organ interactions. Most importantly, the standardized TCM-Organoid Atlas databases which integrates multi-omics and high-resolution imaging has been developed to resolve data fragmentation, that is a limitation absent in Western drug evaluation paradigms. Despite advances in 3D modeling, limitations remain in capturing TCM’s holistic regulatory features. On the one hand, the structure and cell types of organoid models cannot simulate real human environment due to lack of complex cellular components. This is illustrated by the report of Miao et al., whose study showed that the organoid model they developed lacked complex cellular components such as nerve and immune cells, so it could not simulate the functions of lung and gut of adults (Miao et al., 2025). On the other hand, it’s difficult to determine the clinical dose in organoid model due to diversity of physiological status between organoid model and human bodies. For instance, Wu et al. validated the hepatoprotective effect of Schisandra chinensis based on human pluripotent stem cell-derived liver organoid model, while there is still room for improvement in the mode of administration and dosage (Wu et al., 2024).

To overcome these modeling limitations and better emulate TCM’s systemic regulation, integrating artificial intelligence into 3D bioprinting and organoid systems becomes increasingly critical. For example, neural network-based toxicity predictions (Song et al., 2024) inform vMOC experimental designs, whose molecular outputs (e.g., proteomic signatures) would feed back into biomarker discovery. This integration is demonstrated by Buzhong Yiqi Decoction: vMOC validation of its “lung-spleen axis” mechanism identified IL-6/TNF-α as clinical biomarkers, enabling successful repurposing for COPD (Yi-Ling et al., 2020).
